# Supplementary material for: Characterizing cell-type spatial relationships across length scales in spatially resolved omics data
Source: Nat Commun. 2025 Jan 3;16:350. doi: 10.1038/s41467-024-55700-1 (PMC11699133; doi:10.1038/s41467-024-55700-1)
Supplement: Supplementary file 3 — Supplementary Software 1 [file 41467_2024_55700_MOESM3_ESM.zip › CRAWDAD/docs/2_slideseq.html]

2\_slideseq


This vignette will go through analyses to reproduce the results and
figures of the Slide-seq dataset.

```
library(crawdad)
library(tidyverse)
```

```
## Warning: package 'ggplot2' was built under R version 4.3.1

## Warning: package 'dplyr' was built under R version 4.3.1

## Warning: package 'stringr' was built under R version 4.3.1

## ── Attaching core tidyverse packages ──────────────────────── tidyverse 2.0.0 ──
## ✔ dplyr     1.1.4     ✔ readr     2.1.4
## ✔ forcats   1.0.0     ✔ stringr   1.5.1
## ✔ ggplot2   3.5.1     ✔ tibble    3.2.1
## ✔ lubridate 1.9.2     ✔ tidyr     1.3.0
## ✔ purrr     1.0.1     
## ── Conflicts ────────────────────────────────────────── tidyverse_conflicts() ──
## ✖ dplyr::filter() masks stats::filter()
## ✖ dplyr::lag()    masks stats::lag()
## ℹ Use the conflicted package (<http://conflicted.r-lib.org/>) to force all conflicts to become errors
```

```
ncores = 7
```

# Load data

```
data(slide)

## convert to sf
slide <- crawdad:::toSF(pos = slide[,c("x", "y")],
                        cellTypes = slide$celltypes)
```

```
## Warning: 'celltypes' does not have levels. Creating levels from values

## creating `sf` object
```

# Visualize celltypes

```
crawdad::vizEachCluster(cells = slide,
                        coms = as.factor(slide$celltypes),
                        s = 2)
```

```
## TableGrob (4 x 5) "arrange": 19 grobs
##     z     cells    name           grob
## 1   1 (1-1,1-1) arrange gtable[layout]
## 2   2 (1-1,2-2) arrange gtable[layout]
## 3   3 (1-1,3-3) arrange gtable[layout]
## 4   4 (1-1,4-4) arrange gtable[layout]
## 5   5 (1-1,5-5) arrange gtable[layout]
## 6   6 (2-2,1-1) arrange gtable[layout]
## 7   7 (2-2,2-2) arrange gtable[layout]
## 8   8 (2-2,3-3) arrange gtable[layout]
## 9   9 (2-2,4-4) arrange gtable[layout]
## 10 10 (2-2,5-5) arrange gtable[layout]
## 11 11 (3-3,1-1) arrange gtable[layout]
## 12 12 (3-3,2-2) arrange gtable[layout]
## 13 13 (3-3,3-3) arrange gtable[layout]
## 14 14 (3-3,4-4) arrange gtable[layout]
## 15 15 (3-3,5-5) arrange gtable[layout]
## 16 16 (4-4,1-1) arrange gtable[layout]
## 17 17 (4-4,2-2) arrange gtable[layout]
## 18 18 (4-4,3-3) arrange gtable[layout]
## 19 19 (4-4,4-4) arrange gtable[layout]
```

# Make shuffled background

```
scales <- seq(100, 1000, by=100)
```

```
## generate background
shuffle.list <- crawdad:::makeShuffledCells(slide,
                          scales = scales,
                          perms = 3,
                          ncores = ncores,
                          seed = 1,
                          verbose = FALSE)
```

# Run pairwise analysis

```
## find trends, passing background as parameter
results <- crawdad::findTrends(slide,
                        neighDist = 50,
                        shuffleList = shuffle.list,
                        ncores = ncores,
                        verbose = FALSE,
                        returnMeans = FALSE)
```

```
## convert results to data.frame
dat <- crawdad::meltResultsList(results, withPerms = T)
```

# Visualize results

```
## multiple-test correction
ntests <- length(unique(dat$reference)) * length(unique(dat$reference))
psig <- 0.05/ntests
zsig <- round(qnorm(psig/2, lower.tail = F), 2)
```

Summary visualization of CRAWDAD’s multi-scale cell-type spatial
relationship analysis.

```
vizColocDotplot(dat, reorder = TRUE, 
                zSigThresh = zsig, zScoreLimit = zsig*2,
                dotSizes = c(1,13)) +
  theme(legend.position='right',
        axis.text.x = element_text(angle = 45, h = 0))
```

```
## this function will be deprecated, use vizRelationships instead

## Scale for x is already present.
## Adding another scale for x, which will replace the existing scale.
```

Visualize specific trends.

```
dat_filter <- dat %>% 
  filter(reference == 'Purkinje') %>% 
  filter(neighbor == 'Bergmann')
vizTrends(dat_filter, lines = T, withPerms = T, zSigThresh = zsig)
```

```
dat_filter <- dat %>% 
  filter(reference == 'Purkinje') %>% 
  filter(neighbor == 'Oligodendrocytes')
vizTrends(dat_filter, lines = T, withPerms = T, zSigThresh = zsig)
```
